# Avoidance of specific calibration sessions in motor intention recognition for exoskeleton-supported rehabilitation through transfer learning on EEG data

**DOI:** 10.1038/s41598-024-65910-8

**Published:** 2024-07-19

**Authors:** Niklas Kueper, Su Kyoung Kim, Elsa Andrea Kirchner

**Affiliations:** 1 Robotics Innovation Center, German Research Center for Artificial Intelligence (DFKI), 28359 Bremen, Germany; 2 Institute of Medical Technology Systems, University of Duisburg-Essen, 47057 Duisburg, Germany

**Keywords:** EEG, Movement prediction, Rehabilitation, Robot-assisted therapy, BCI, Transfer learning, Computational neuroscience, Data acquisition, Data processing, Machine learning, Software, Neurological disorders, Cardiovascular diseases, Computational biology and bioinformatics, Neuroscience, Diseases, Health care, Medical research, Neurology, Engineering, Biomedical engineering

## Abstract

Exoskeleton-based support for patients requires the learning of individual machine-learning models to recognize movement intentions of patients based on the electroencephalogram (EEG). A major issue in EEG-based movement intention recognition is the long calibration time required to train a model. In this paper, we propose a transfer learning approach that eliminates the need for a calibration session. This approach is validated on healthy subjects in this study. We will use the proposed approach in our future rehabilitation application, where the movement intention of the affected arm of a patient can be inferred from the EEG data recorded during bilateral arm movements enabled by the exoskeleton mirroring arm movements from the unaffected to the affected arm. For the initial evaluation, we compared two trained models for predicting unilateral and bilateral movement intentions without applying a classifier transfer. For the main evaluation, we predicted unilateral movement intentions without a calibration session by transferring the classifier trained on data from bilateral movement intentions. Our results showed that the classification performance for the transfer case was comparable to that in the non-transfer case, even with only 4 or 8 EEG channels. Our results contribute to robotic rehabilitation by eliminating the need for a calibration session, since EEG data for training is recorded during the rehabilitation session, and only a small number of EEG channels are required for model training.

## Introduction

With the demographic change, the cost of stroke in 2017 was €60 billion in the 32 European countries alone^1^. Effective rehabilitation for stroke patients is needed. To enable more effective sensorimotor rehabilitation therapy, traditional physiotherapy can be combined with robot-supported therapy^2–4^. Such approaches reduce costs, increase the efficiency of therapy, and relieve the burden on the therapists by enabling high repetitions in interactive and self-initiated therapy as well as by extending therapy options^5^. It was shown that patients receiving intensive peer mentoring during and after rehabilitation had greater gains in self-efficacy^6^, which is highly important in rehabilitation^7^ and decreases the time for unplanned rehospitalizations^6^. The application of new robotic technologies, are expected to reduce the total disease burden by 6 to 10 percent by 2040^8^. Active exoskeletons^9^ are commonly used for assistance in daily living (ADL)^10–12^ as well as for rehabilitation therapies, and have shown to be effective in neuromotor rehabilitation, especially after stroke^13–15^.

**Figure 1:**
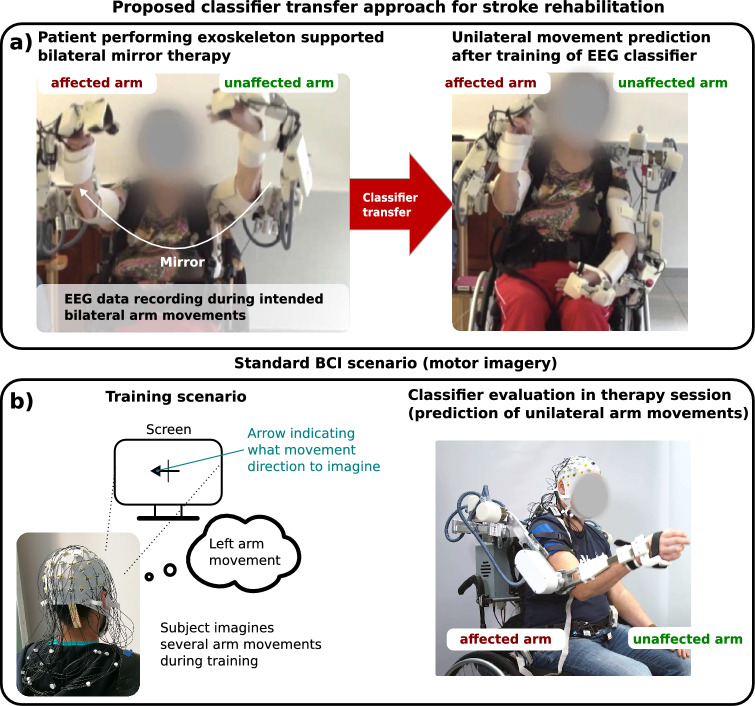
Comparison between the proposed classifier transfer approach for stroke rehabilitation (see a)) and a standard MI scenario used in rehabilitation (see b)). In (**a**) a patient, exercising in the exoskeleton’s mirror mode is shown while EEG data is recorded. The classifier is transferred after training to predict the movement intentions of the affected arm. In (**b**) a standard MI training scenario with an evaluation of the classifier for stroke rehabilitation is shown. The images are used from a supporting video (source: https://www.youtube.com/watch?v=dCn1ktzbpZ8).

For individualized support of patients, learning of individual machine-learning models from human data is required, to classify such physiological data online. For example, to provide support when needed^16–18^, physiological measurements such as the electromyogram (EMG), which can, for example, be recorded from a healthy leg to control a disabled leg using an exoskeleton^19^, or the electroencephalogram (EEG) which allows conclusions to be drawn about a user’s movement intention, is of great importance for successful neurorehabilitation^13,14^. In particular, EEG can be used to infer movement intentions^9,20^, for example, where a patient wants to move^21,22^. There are many examples of how human EEG can be used to control exoskeletons using a brain-computer interface (BCI)^13,14,21–25^. However, BCIs are often not used to decode brain activity that correlates with the brain processes that control movement intention, planning, and execution; instead, other brain signals, such as the activity evoked in the visual cortex by flickering light, known as steady-state visual evoked potentials (SSVEP) are used^21,22,26^. Such BCI’s artificially use the patient’s EEG as a control input. To bridge the gap between brain and body caused by brain injury and to promote rehabilitation, such an approach is not the preferred one. Instead, brain activity that drives movement intention, planning, and execution should be used as a natural or intrinsic bridge between the brain and the body^27,28^. Such an approach will use both, the physiological data that directly encodes the human’s intention and the autonomous capabilities of the robotic system, i.e., an exoskeleton. Since the patients, especially severely affected stroke patients, are not able to move their affected limb, motor imagery (MI) is often used to generate motor-related patterns in the EEG (e.g. see Fig. 1b). This means the patient only imagines the movements repeatedly instead of attempting actual movement executions. But still, the evoked MI activity in the EEG is comparable to the activity evoked by movement executions^29–31^. However, in the field of BCIs, long training sessions are often required to record a large amount of training data. Thus, such intensive training sessions require the patient to imagine movements several times. This goes along with two problems: (1) It is difficult to monitor and ensure a good vividness of the imagined movements^32,33^; (2) such training sessions are very tiresome and do not promote therapy while using time that could otherwise be used for an effective therapy session. Even if a patient can complete such long training sessions, this is not desirable since all time available should be used for therapy in the early post-stroke period in which the brain is very plastic^34^. Waste of time in this very sensitive period by plainly recording data to train a classifier must be avoided as much as possible.

To address this issue, transfer learning (TL) can be applied in BCI applications to reduce or even avoid calibration sessions. This can be achieved by using prior knowledge or data that does not originate from the target session, subject, or even measurement device or task^35^. TL has recently proven it’s potential to improve classification performance and reduce calibration times in several investigations^36–40^. By applying such a TL approach, the amount of required data and the duration of training sessions that are not beneficial for the patient’s rehabilitation can be reduced. However, in most cases, the patient still needs to participate in training or calibration sessions that do not directly serve rehabilitation. Hence, besides cross-session as well as cross-subject paradigms, cross-task TL approaches are investigated for a complete avoidance of calibration sessions. Such cross-task approaches even enable learning in the first place if labeled EEG data is only available from a similar task. Choosing a similar task that is part of the therapy session and by applying a cross-task approach would make it possible for patients to no longer need to participate in training sessions to train an ML model for decoding EEG data. However, in comparison, cross-task classification approaches based on EEG data and TL have been less comprehensively investigated^35^.

Nevertheless, across different fields of BCI research, transfer approaches for cross-task EEG classification have been proposed. In our previous work, we showed that a classifier trained on EEG data from an observation scenario could be transferred to detect the erroneous behavior of a robot during an interaction scenario^41–44^. Therefore, the elicted event-related potential (ERP), namely the error-related potential (ErrP), could be classified in the transfer case although the tasks and ERP shapes differed between training and testing. The transferability of a classifier for the detection of errors across tasks was also shown in^45^, where a deep convolutional neural network was used to detect errors for two different error paradigms from intracranial EEG data. Another application of a classifier transfer is the detection of target and *missed target *events from EEG while the classifier was trained on EEG data evoked by target and *standard* events (oddball paradigm) to enhance the amount of available training data and, hence, to enable classification of EEG trials evoked during recognition of targets and the failure of recognition of those^46–48^. Further examples of applied cross-task EEG classification can be found in the literature in the area of workload recognition^49–51^, where the performed workload task differed between training and testing a classifier or model. For example in^49^, a domain adaptation approach was applied that improved the workload classification performance for the transfer case compared to a non-transfer case. Besides cross-task EEG classification, where a classifier is strictly trained on one task and tested on another task, fusion approaches were applied in which data from different tasks were combined for training a classifier^52,53^.

In this work, we focus on how to generate training data for an EEG-based intention recognition to guide support using an active exoskeleton^54^ for unilateral arm movements of the affected arm after stroke. To obtain a natural bridge, EEG data related to the execution of movements is used instead of EEG evoked by MI. With our study on healthy subjects, we want to show, that our envisioned approach to train a classifier on EEG data recorded during robot assisted mirror therapy of patients, that enables bilateral movements, can be used to predict unilateral arm movements. In more detail, our approach will allow to train a classifier during a therapy session that does not require intention recognition from EEG activity but makes use of the intelligence of the robotic system to timely map movement intention to movement execution. This is done by making use of the exoskeleton’s mirror mode in which a movement of the unaffected arm is transferred to the affected arm by the exoskeleton (see Fig. 1a)^54^. Hence the exoskeleton supports mirrored dual arm movements intended by the patient. While the patient is exercising in this mode, EEG data can be recorded. The recorded EEG contains activities associated with the planning and execution of bilateral arm movements and can be used to train a classifier to infer the movement intention of both arms. The usability of such a classifier cross-task transfer approach to infer the motion intention of the (supposed to be the) affected arm alone will be investigated in our study presented here. To this end, we evaluate it’s principle feasibility by conducting a study with healthy subjects and report and discuss the results here. Our approach eliminates any exclusive time spent by the patient for only generating training data, as data collection is integrated into the therapy training itself. To our knowledge, the transferability of a classifier between bilateral and unilateral movements (upper body reaching movement tasks), especially conceptualized to support rehabilitation therapy, has not been proposed or investigated so far.

The rest of the paper is structured as follows. Section *Methods* provides detailed information about our proposed classifier transfer approach to support patients in future rehabilitation sessions as well as the conducted study and performed evaluations. This includes detailed information about the processing of the recorded data and evaluation of the proposed cross-task classifier transfer approach under a varying number of used EEG channels. Afterward, the results of our evaluations are described in the corresponding *Results* section. In section *Discussion* the main findings of our evaluations regarding the proposed classifier transfer approach and its implications for our future work are discussed in detail. Finally, section *Conclusion* provides a summary of this work.

## Methods

### Proposed future therapy approach

Our proposed approach is intended to support (mainly strongly affected) stroke patients in therapy sessions supported by an active exoskeleton, where the unaffected arm of the patient will control the affected arm with the help of the exoskeleton in a so-called mirror mode. The exoskeleton does execute mirrored movements to enable bilateral movement support. This means the movement of the unaffected arm is mirrored to the affected arm while the task for the patient is still to move both arms to induce bilateral motor activity in both hemispheres. This also means that these executed bilateral movements are already part of the therapy so it is not considered as an additional calibration session. However, the exoskeleton is not able to support the affected arm based on the movement intention of the patient. Due to this, we want to predict the movement intention of the affected arm by analysis and decoding of the patient’s EEG. For training a classifier for this task, we use the EEG recorded during mirror mode therapy as explained before. By this cross-task transfer, we completely avoid calibration sessions for using the BCI.

### Proposed classifier training method

To train an EEG classifier to only predict movement intentions for the affected arm of stroke patients in the future rehabilitation sessions, a two-step concept was developed. It has to be noted that in this work with healthy subjects, we assumed the right arm of the subjects to be the affected one and the left arm to be the unaffected one. In the first step, the EEG classifier is trained during the execution of bilateral arm movements (in a mirror mode rehabilitation session). The onsets of the bilateral movements are inferred from the unaffected arm to generate reliable movement onset labels to train the classifier. In the second step, the classifier is transferred to predict unilateral movements of the affected arm. The transfer approach consists of training on EEG data derived from bilateral movement executions and applying a custom EEG-channel selection to improve the transferability of the classifier by data adaptation. Since the LRP (Lateralized Readiness Potential)^55,56^, which is associated with movement planning, can be observed from EEG-channels of the motor cortex side contralateral to the moved upper limb^57^, we focus on the differences and similarities in the EEG data between bilateral and unilateral movement intentions. Therefore, we customized our selection of EEG channels for the data processing, that is related to the planning of unilateral movements with the affected limb. Hence, the abilities of the unaffected limb are used to generate reliable training labels and the classifier can be custom-trained on the provided EEG data, containing information about the movement intentions of the affected arm. The proposed method was evaluated by conducting experiments, involving bilateral and unilateral movement tasks, executed by healthy subjects.

**Figure 2:**
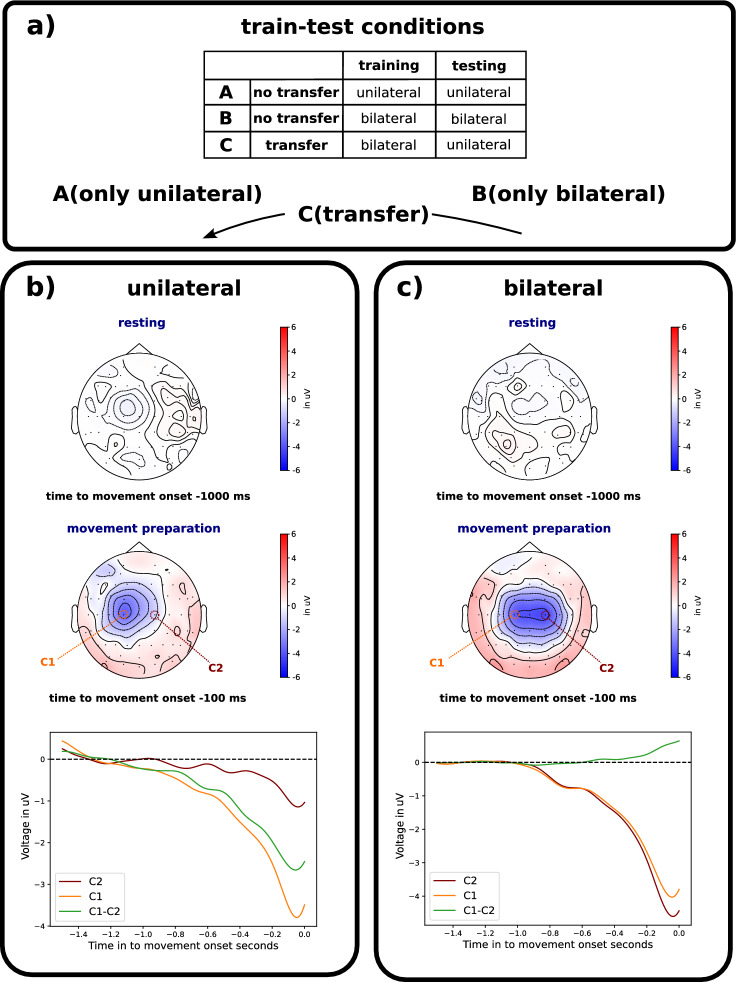
Concept for the evaluation of the classifier and the results of the ERP analysis (topography and grand average ERPs). Three train-test conditions (A, B, C) were selected for the evaluation of the classifier and shown in (**a**). The ERP analysis shown in (**b**) and (**c**) was performed on the data of the unilateral and bilateral movement tasks. For the ERP analysis, the data was band-pass filtered ($0.1-4\,\hbox {Hz}$), eye blinks were removed by manual exclusion of ICA components, and segmented into epochs from $-1.5\,\hbox {s}$ to $0\,\hbox {s}$ based on the movement onset. Before averaging epochs, a baseline correction of $-1.5\,\hbox {s}$ to $-1\,\hbox {s}$ was applied based on the movement onset. A total of 960 epochs across all subjects were used to calculate the grand average of ERPs for both movement tasks. The grand average ERPs and topography were visualized for the two movement tasks: unilateral in (**b**) and bilateral in (**c**).

The training and testing conditions of the evaluation are illustrated in Fig. 2a): *train-test* conditions: (A) *unilateral-unilateral* (no transfer), (B) *bilateral-bilateral* (no transfer), and (C) *bilateral-unilateral* (cross-task transfer). Condition C is our target condition, i.e., cross-task transfer takes place. The classifier is trained on EEG evoked by bilateral arm movements and transferred to infer intended unilateral arm movements. Condition A (training and testing on EEG evoked by movements of the supposed to be affected arm) and Condition B are only used to compare how well a classifier performs without transfer compared to the cross-task transfer (Condition C). Moreover, for our envisioned application, the most relevant performance differences are between condition C (cross-task transfer from bilateral to unilateral) and condition A (unilateral-unilateral, no transfer), where training and testing were evaluated on EEG activity recorded from the hemisphere contralateral to the moved arm.

### Experimental setup and procedure

**Figure 3:**
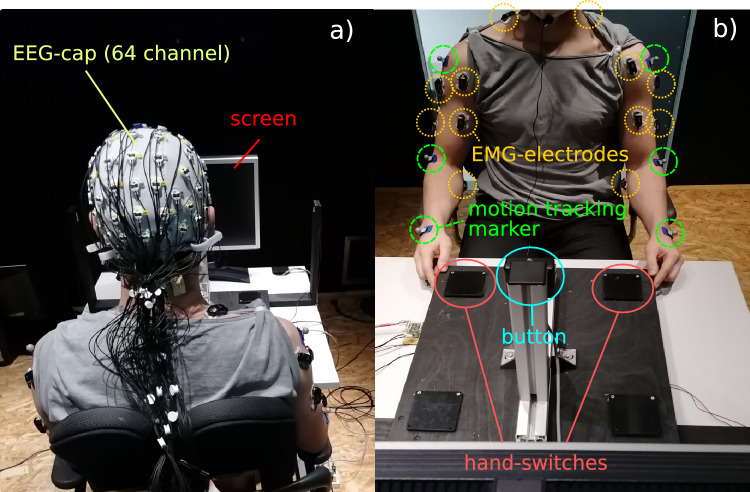
Experimental setup of the study. In (**a**) a subject is shown sitting in front of a screen wearing an EEG cap with 64 electrodes. In (**b**) the custom build experimental board including hand-switches (orange) and a button (blue) as well as the placed EMG-sensors (yellow) and motion tracking marker (green) are illustrated.

Eight healthy subjects (4 male, 4 female) at the age of $25.5 \pm 4.0$ years participated in our study. The study was approved by the ethics committee of the University of Bielefeld according to the guidelines of the German Society for Psychology and the Professional Association of German Psychologists and all subjects gave their written informed consent to participate in the study. Only healthy right-handed subjects with no history of neurological or muscular diseases were recruited for the experiments. All subjects were advised to be well-rested for the experiment. The experiment setup is illustrated in Fig. 3. The subjects were seated in a comfortable chair inside a shielded cabin. In front of the subjects a custom-built board, including hand-switches and a button were placed on a table. The subjects were asked to perform a reaching task, by pressing the button with their thumb. The button was placed at a height of approximately $25\,\hbox {cm}$ and at a distance of $30\,\hbox {cm}$ away from the resting position. The resting position was defined by the hand switches, where the subjects were asked to place their hands during the resting period. The position of the button was adjusted to the arm length of the subjects. The start and endpoint of the movements were standardized by ensuring a 90-degree forearm-upper arm angle at rest and 0 degree when pressing the button.

Two types of movement tasks were conducted in the experiment (see Fig. 2b, c): 1. unilateral reaching movements and 2. bilateral reaching movements. The sequence of the two task types was varied between subjects (counterbalanced) to neutralize possible learning effects. For the unilateral task, only the dominant right arm was moved whereas in the bilateral task, a synchronous movement of both arms (both thumbs pressing the button) was executed. Each task included 3 sets of 40 self-initiated movements. Therefore, each subject performed a total of 120 trials for each task. Each trial consisted of a resting period of at least 5 s followed by a self-initiated and self-paced reaching movement. Trials with a resting period under 5 s were excluded from the evaluation and an error symbol was presented on a monitor for a duration of $200\,\hbox {ms}$. The error symbol consisted of a fixation cross that turned from a green to a red background color. During the whole experiment, a fixation cross with a green background was continuously shown on the monitor. After each set, the subjects were asked to relax for at least 5 min to avoid any fatigue. The whole experiment was designed and controlled by using the Presentation software [Neurobehavioral Systems, Inc., Albany, USA].

### Data acquisition

EEG data was recorded using a LiveAmp64 amplifier and an actiCap montage with 64 active electrodes [Brain Products GmbH, Munich, Germany]. The electrodes were located according to the extended 10–20 system with FCz as a reference electrode. All impedances were kept below a threshold of $5\,\hbox {k}\Omega$ and were controlled after each measurement set. The data was acquired at a sampling rate of $500\,\hbox {Hz}$ and prefiltered by the measurement device to a bandwidth of $0.1-131\,\hbox {Hz}$. To avoid possible artifacts during the recording, the subjects were asked to avoid head and eye movements as far as possible.

EMG signals were recorded bipolar (Ag/AgCl electrodes) by a WavePlus wireless system and picoEMG sensors by Cometa [Cometa srl., Barregio, Italy]. The EMG was sampled at $2000\,\hbox {Hz}$ and reduced to a bandwidth of 10–$500\,\hbox {Hz}$ by filters of the measurement device. The signals were recorded from 8 muscles for the right and left side of the body which were: M. biceps brachii medial, M. triceps brachii medial M. triceps brachii lateral, M. deltoideus lateral, M. deltoideus anterior, M. deltoideus posterior, M. trapezius pars descendens (upper trapezius) and M. flexor carpi radialis. The skin was prepared with alcohol and electrodes were placed according to anatomical landmarks^58^.

To mark the physical movement onsets, an infrared motion tracking system [Qualisys AB, Gothenburg, Sweden] was used in addition to the mechanical hand-switches. In total, 4 motion tracking cameras (Oqus $300+$) were placed in the shielded cabin to record motion data. To track the motions, 3 reflecting markers were placed on the back of the hand, the elbow (next to the lateral epicondyle) and the deltoideus (muscle belly) on each side of the body. The motion tracking data was acquired at a sampling rate of $500\,\hbox {Hz}$.

All events during the experiments, such as pressing/releasing the hand switches and the button, as well as invalid trials (shown error symbols), were tracked by the EEG system. Additionally, the start and stop of the recordings of each measurement system were recorded by the trigger channels of the EEG system to synchronize all the data in the offline analysis.

### Estimation of physical movement onset

For estimating the physical (ground truth) movement onset, the position data tracked by the motion capture system were analyzed and processed in an offline evaluation. Since the executed reaching tasks consisted of moving the hand from a resting position towards the button, the data from the reflective marker of the moved hand was used for the estimation. Note that in a later rehabilitation session, movement onsets will be detected by the exoskeleton^25,59^. Since only healthy subjects participated in the study, the right arm was assumed to be the affected arm, while the left arm was assumed to be unaffected as stated above. Therefore, for bilateral movements only the position data of the left hand was selected for estimating the ground truth movement onset.

In the first processing step, the EEG and motion capture data were synchronized. Afterward, the position data was re-initialized to the resting position by subtracting the mean position data, calculated from the first second (resting period) of each experiment. In the next step, the absolute distance to the resting position was calculated by computing the euclidean distance from the three-dimensional position data. Additionally, the velocity of the hand was calculated for each timepoint by taking the difference between two consecutive samples of the euclidean distance. The velocity was filtered by a lowpass filter with a cutoff frequency of $4\,\hbox {Hz}$ (butterworth, 4. order) and normalized to the maximum value of the current trial. The distance and velocity were then combined by multiplication in order to provide an exact estimate of the movement onset. This procedure was chosen to calculate the onset, independently of small position fluctuations (producing high-speed values) or slight variations of the resting position between trials.

Starting from the movement period towards the resting period, it was searched backward for a data point with a magnitude below a defined threshold. The search started at the time when the mechanical hand switch was released since this is assumed to be the movement onset plus the mechanical delay of the device. The threshold was set to $0.6\,\hbox {mm}$ and specified concerning the resolution of the motion tracking system after calibration. The movement onsets were marked in the EEG data.

### Channel selection and reduction

In order to provide proper transferability of the classifier, we custom selected EEG channels by means of the knowledge about the surface distribution of relevant EEG activity with respect to individual EEG channels. Since the LRP can be observed in the hemisphere contralateral to the side of the moved limb (right arm), we custom-selected channels for the classifications that were located on the left hemisphere, especially in the area of the motor cortex. With this approach, we aim to enhance the transferability of the classifier, which is trained on evoked EEG potentials from bilateral movement planning to predict unilateral movement intentions by selecting EEG channels related to right arm movements.

**Figure 4:**
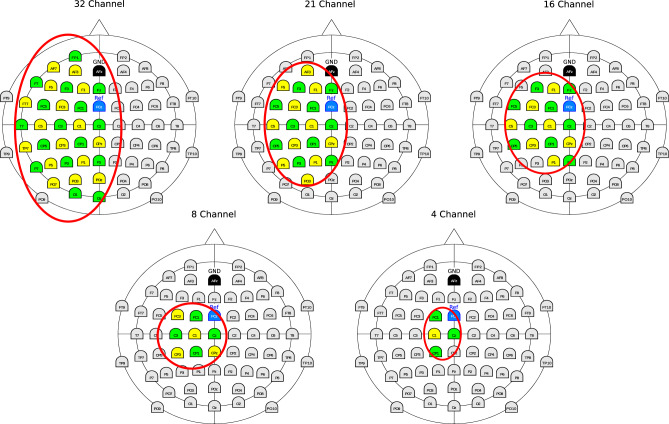
Custom selected channels from the left hemisphere. Channels used for the study are marked by a red circle. The first 32 channels of the 64-channel cap layout are marked in Green, whereas the second 32 channels are marked in Yellow (combined to a total of 64 EEG channels).

Besides enhancing the performance of the transferred classifier, we also aim to reduce the number of channels to provide an approach that is feasible to be used with persons suffering from stroke. Therefore, we systematically reduced the number of EEG channels used for the prediction of movement intentions in order to reduce the preparation effort in a real rehabilitation session. Due to this, we evaluated the use of 32, 21, 16, 8, and 4 custom-selected channels to predict movement intentions. For the different numbers of channels, the selection was made considering the C1 channel as a center of EEG activity related to movement planning, with the other channels located around it. Therefore, by reducing the number of channels the area covered by the electrodes around this center was further reduced in size. The specified EEG channels for the custom selection are illustrated in Fig. 4.

**Figure 5:**
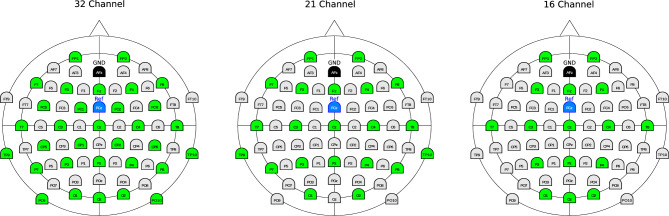
Standard channel constellation based on the extended 10–20 system. The channels used for this study are marked in green.

In order to evaluate the relevance of custom channel selection, we further compared the custom selection to standard electrode constellations based on the extended 10–20 system. Since such a standard constellation comprises at least 16 EEG channels, we evaluated and compared 32, 21, and 16 channels for the standard constellation as a baseline for our custom channel selection. The standard channel constellations for the different numbers of channels are illustrated in Fig. 5.

### EEG processing and classification

For the processing and classification of the EEG signals, the signal processing and classification platform pySPACE^60^ was used. A previously developed machine learning pipeline^20^, specialized to detect the LRP, was adopted.

#### Preprocessing and windowing

The EEG signals were processed window-wise by cutting out overlapping windows with a length of $1\,\hbox {s}$ and a stepsize of $0.05\,\hbox {s}$. For each trial, a total of 81 windows, starting from window $[-5.00, -4.00]\,\hbox {s}$ to $[-1.00, 0.00]\,\hbox {s}$ were cut out with respect to the labeled physical movement onset at $0\,\hbox {s}$.

First, a subset of EEG channels corresponding to the evaluated channel selection methods was included in the next processing steps (see Channel Selection and Reduction). Subsequently, the data were standardized channel-wise (zero mean, SD of one) and decimated to $20\,\hbox {Hz}$. Next, a FFT bandpass filter with a passband of 0.1–$4.0\,\hbox {Hz}$ was applied.

#### Feature extraction and classification

The channel dimension was reduced by applying an xDAWN spatial filter^61^ with 4 remaining pseudo-channels, which was designed to enhance event-related potentials. Afterwards, the last 4 samples of each window, that correspond to the last $0.2\,\hbox {s}$, were extracted as time domain features. Therefore, a total of 16 features were extracted for each window. The features were then normalized by applying a Gaussian feature normalization (zero mean, variance one). After feature extraction, an SVM with L1-norm regularization was trained for a binary classification task. The class labels were NoLRP (resting) and LRP (movement intention). To train the classifier, 2 out of 3 recorded measurement sets (80 movement trials) were used as training data and the remaining set (40 movement trials) was separated for testing (for more details see section *Performance Evaluation and Metrics)*. The complexity parameter (hyperparameter) of the SVM was optimized by applying a grid search with 7 equal-spaced values in a range of $10^{-6}$–$10^{0}$ and using a five-fold cross validation on the training dataset to obtain the optimal hyperparameter. After obtaining the optimal complexity value, the SVM was trained on the whole training dataset. The class weights of the SVM were set to a ratio of 1:2 (NoLRP:LRP). The windows $[-1.10, -0.10]\,\hbox {s}$ and $[-1.00, 0.00]\,\hbox {s}$ were used as training instances of the LRP class and the windows $[-3.05, -2.05]\,\hbox {s}$, $[-3.25, -2.25]\,\hbox {s}$ and $[-3.50, -2.50]\,\hbox {s}$ were selected as training instances of the NoLRP class. After training, the classifier was used to predict all windows of a separate test set. This was done to simulate a real online application scenario, where a classifier continuously determines whether EEG windows correspond to a resting period or a period of movement intention. The SVM scores were then transformed into a probability by using Platts sigmoid function^62^. A probability greater than 0.5 corresponded to a detected intention to move (LRP class); otherwise, a rest period (NoLRP class) was detected.

#### Performance evaluation and metrics

Since the class ratios (NoLRP:LRP) are unbalanced for the continuous detection of movement intentions (longer resting periods than movement planning) the balanced accuracy (BA) was used as a performance metric. The balanced accuracy calculates the performance concerning the individual class rates for both classes and is defined as the mean of the true negative rate (TNR) and the true positive rate (TPR). During the evaluation, care was taken that the TNR and TPR for each classification result were not imbalanced to avoid a disbalance or bias between the prediction of the NoLRP and LRP classes.

To emulate an online application scenario, the classifier was evaluated by creating set-wise train and test pairs. As mentioned above, for each condition, 2 measurement sets were used for training and the remaining set was used as a test set to evaluate the performance (leave one set out validation). Therefore, for each condition, a total of 24 performance results were produced due to 3 train/test permutations for all 8 subjects.

To evaluate the performance results with respect to the characteristics of the LRP, a relabelling technique was applied to the classification outcome in order to generate ground truth labels for performance evaluation. Since the individual planning and execution of a movement, for example, depending on the waiting time, is affecting the temporal characteristics of the LRP^63^, a variability between single trials must be considered. Since the actual start of the movement planning remains unknown, ground truth labels of the windows were computed based on the classification outcome for each individual trial considering an online application. In the following, the procedure is described in detail.

**Figure 6:**
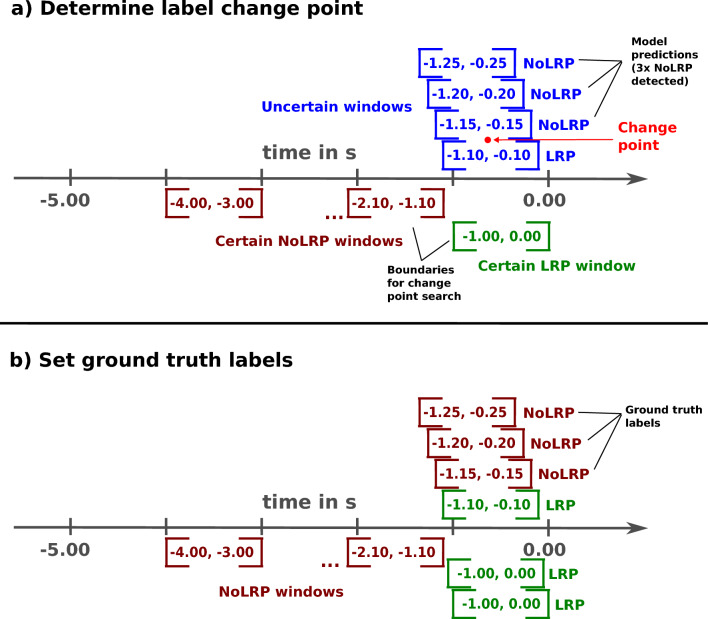
Illustration of the relabeling technique. In (**a**) the determination of the label change point between two consecutive windows is shown while in (**b**) the ground truth label after applying the method is illustrated.

First, a change point of classes (class boundaries) was computed, which gives an estimate of a started movement planning phase after the resting period and therefore the starting point of the LRP class in time. This change point was defined as a point between two consecutive windows that lies within an interval between window $[-2.00, -1.00]\,\hbox {s}$ to window $[-1.00, 0.00]\,\hbox {s}$. This is the range where movement planning is to be expected when continuously classifying windows in an online application scenario. The windows at the boundary of the defined interval correspond to windows where the true label is known with high certainty for the NoLRP ($[-2.00, -1.00]\,\hbox {s}$) and LRP ($[-1.00, 0.00]\,\hbox {s}$) class. The choice of the class boundaries was also discussed in our previous work in^20^. If three consecutive NoLRP windows were counted backward in time (starting from window $[-1.00, 0.00]\,\hbox {s}$ backward) within this range, the label change point was detected and the labels of all windows before this point were set to the NoLRP class and past this point to the LRP class. In case no change point was found inside this range, all windows within this range were defined as instances of the LRP class corresponding to a detected long movement planning phase. However, for windows where the true label is known (from the experimental design), the class label remained fixed for each movement trial. Therefore, all windows before window $[-2.00, -1.00]\,\hbox {s}$ were always instances of the NoLRP class while window $[-1.00, 0.00]\,\hbox {s}$ was always an instance of the LRP class. In conclusion, this technique was used to provide ground truth labels for each sliding window based on the nature of the LRP under predefined constraints where the detection of a movement intention was allowed. To illustrate the procedure, the applied method is shown in Fig. 6.

### Statistical analysis

**Figure 7:**
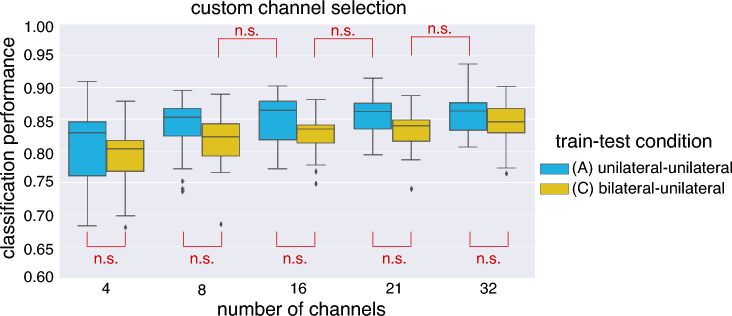
Transfer effect: classification performance between both *train-test* conditions: (A) no transfer (*unilateral-unilateral*) and (C) transfer (*bilateral-unilateral*). Details for *train-test* conditions, see Fig. 2a). The *n.s.* stands for no significant difference.

**Figure 8:**
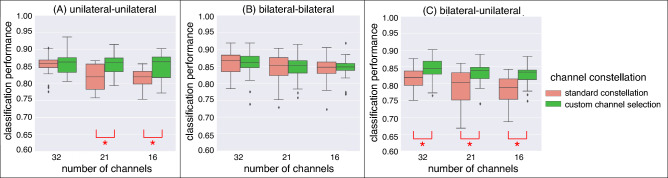
Effect of electrode distribution: classification performance between standard constellation and custom channel selection for all *train-test* conditions (**A**, **B**, **C**). Details for *train-test* conditions, see Fig. 2a). The $*$ stands for a significant difference.

The classification performances were analyzed by two-way repeated measures ANOVA with *number of channels* and *train-test condition* (Fig. 2a) as within-subjects factors to investigate the effect of cross-task *transfer* depending on the number of channels: *transfer* vs. *no transfer* (see Fig. 7). Additionally, we performed two-way repeated measures ANOVA with *channel constellation* and *train-test condition* (Fig. 2a) as within-subjects factors to compare both a standard constellation and custom channel selection for each *train-test condition* (see Fig. 8).

### Ethical approval

The conducted study was examined and found to be harmless by the University of Bielefeld according to the ethical guidelines of the German Society for Psychology and the Professional Association of German Psychologists.

## Results

Figure 7 shows the comparison of the classification performance between the following conditions: (A) *unilateral-unilateral* (no transfer; baseline) and (C) *bilateral-unilateral* (transfer). As stated before for our envisioned application conditions A and C are most relevant with respect to the question of what the performance differences are between cross-task transfer from bilateral to unilateral (condition C) and no transfer in which training and testing takes place on EEG activity recorded from the hemisphere contralateral to the moved arm (condition A). We found no significant differences between both *train-test* conditions (A vs. C) for all channel number setups (32, 21, 16, 8, 4). That means that the classification performance was slightly reduced for the *transfer* case but did not differ significantly from the *no transfer* case. In addition, we found no significant differences between the other comparison pairs of the *train-test* conditions, i.e., (A) versus (B) as well as (B) versus (C), for all setups of channel numbers (32, 21, 16, 8, 4). Note that the evaluation design was illustrated in Fig. 2a, i.e., three types of *train-test* conditions (A, B, C).

Figure 8 shows the classification performance between both types of channel constellations: (1) standard constellations vs. (2) custom channel selection. The custom channel selection improved the classification performance. This was evident for the case *transfer* [standard constellations vs. custom channel constellations: *n.s.* for all setups of channels, see *train-test* condition (C) in Figure 8]. The case *no transfer* also benefited from the selection of custom channels when the number of channels was reduced (see *train-test* condition (A) in Fig. 8). However, when EEGs from bilateral movements were used for training and testing, we found no differences between both channel constellations, although, in the custom channel selection, only channels from the left hemisphere were used. Moreover, the classification performance was not affected by channel reduction (see, *train-test* condition (B) in Fig. 8).

## Discussion

In this work, we proposed a novel approach to generate labeled EEG data from bilateral movement executions to train a classifier to predict unilateral movement intentions with high performance. The results show that unilateral movement intentions can be predicted with a balanced accuracy up to 0.845 (for 32 channel, see Fig. 7) using the proposed approach for transferring the classifier. This implies that recorded EEG data from a bilateral interaction session can be used to predict unilateral movement intentions with high performance. Additionally, the results showed that there were no significant differences between classification performances of the *no-transfer* condition *unilateral-unilateral* (A) and the *cross-task transfer* condition *bilateral-unilateral* (C) using the custom channel selection in case that a reduced number of channels was used for the classifications for the cross-task transfer condition (C) (see Fig. 7).

Moreover, we found that a custom selection of EEG channels outperformed the use of a standard channel constellation for training and transferring the EEG classifier (see Fig. 8). Therefore, the custom channel selection outperformed the standard channel constellation, especially for the transfer case. These results indicate, that the custom channel selection allows the possibility to provide a proper transferability of the classifier to predict unilateral movements although only bilateral movements were executed in the training session.

In summary, our proposed cross-task transfer approach yields comparable performances to the no-transfer case for a unilateral movement prediction task based on EEG data. These results conducted with healthy subjects suggest that a cross-task transfer is possible and might be used in the future to transfer a ML model trained during an exoskeleton-supported mirror mode rehabilitation session to trigger movements support of an affected arm of a patient by the exoskeleton. A transfer of the results between healthy subjects and patients seems promising since the classifier is trained on the data evoked in the hemisphere that is affected by stroke. Hence, the classifier can learn the affected pattern. The ability of a classifier to learn to infer movement intention from affected brain signals is supported by our own preliminary measurements with a small sample of stroke patients. These preliminary results indicate that movement intentions can be inferred with a performance comparable to that of healthy subjects although brain activity patterns are different due to the cerebral damage after stroke. Also, literature shows, that although cerebral damage might make it more challenging to detect movement intentions from the EEG with actual stroke patients due to possible differences in active cortex regions or signal amplitude^64^ as well as additional artifact contamination of the signals^65^ the detection of movement intentions from the EEG after stroke is feasible^66,67^. Hence, being aware that the neural activity pattern may be altered after stroke, we think that the proposed approach can be successfully transferred to patients since the altered EEG pattern will be learned by the classifier from actually evoked movement preparations in the EEG of the paretic hemisphere as well as the surrounding motor area. Further studies with stroke patients are planned to answer this open question of our proposed new approach.

As expected, the results of our analysis of the effect of the number of electrodes showed, that the performance of the classifier systematically decreases with the number of used channels for all conditions. Nevertheless, the results show that the number of channels can be reduced, for example, from 32 to 21 channels without a significant performance loss in case of classifier transfer (condition C in Fig. 7). Hence, a subset of channels covering to some extent relevant brain regions provide sufficiently relevant features for the detection of movement intentions. Even more interesting was, that we did not find significant differences between the cross-task transfer condition *bilateral-unilateral* (C) and no transfer baseline condition *unilateral-unilateral* (A) even though the number of channels was reduced for example from 32 to 21 included channels (see Fig. 7). This strongly motivates the applicability of the proposed approach even more, due to a clear reduction of preparation time when using a reduced number of EEG channels. Nevertheless, the channels must be carefully selected and were specifically chosen in the conducted study depending on the motion task to allow channel reduction without much loss in performance. Therefore, the same number and selection of EEG channels may not be adequate for a different movement task (e.g., hand movements) and an alternative manual or automatic technique can be required when reducing the number of used EEG channels.

## Conclusion

We proposed a novel approach to train an EEG classifier on EEG data recorded during bilateral reaching movements supported by an active exoskeleton that is afterward transferred to predict unilateral reaching movements. The cross-task classifier transfer was supported by our knowledge-based selection of EEG channels. The approach was evaluated with data from healthy subjects recorded in the conducted study. It was shown that the proposed transfer approach can predict unilateral movement intentions with a high performance although the classifier is trained only on EEG data recorded during bilateral movements and even when using only a small amount of channels.

Due to the promising results, we are planning further investigations with stroke patients to evaluate the proposed approach to improve stroke rehabilitation. In future work, we plan to use our approach in robot-supported rehabilitation using an upper body exoskeleton. In such an application bilateral movements of a hemiplegic patient can be detected from movement onsets of the unaffected arm while the assistive robotic device moves the affected arm synchronously with the unaffected arm (mirror mode). Hence, rehabilitation therapy can take place while EEGs are recorded and automatically labeled to generate labeled training data. After training a classifier on this data, unilateral movement intentions of the affected arm can be detected from it and supported by an exoskeleton. However, we assume that in the case of patients suffering from stroke channel selection might have to be adapted. To this end, an automated approach considering the type and effect of the lesion would be preferable.

We believe that our novel, robot-assisted rehabilitation approach can improve future rehabilitation therapy in coping with the effects of demographic changes such as the increase in life expectancy and the accompanying need for more support for the aging population.

## Data Availability

The datasets generated and/or analyzed during the current study are available in the following Zenodo repository: https://doi.org/10.5281/zenodo.10229480.
